# Hypoxia Potentiates Anabolic Effects of Exogenous Hyaluronic Acid in Rat Articular Cartilage

**DOI:** 10.3390/ijms17071013

**Published:** 2016-06-25

**Authors:** Shohei Ichimaru, Shuji Nakagawa, Yuji Arai, Tsunao Kishida, Masaharu Shin-Ya, Kuniaki Honjo, Shinji Tsuchida, Hiroaki Inoue, Hiroyoshi Fujiwara, Seiji Shimomura, Osam Mazda, Toshikazu Kubo

**Affiliations:** 1 Department of Orthopaedics, Graduate School of Medical Science, Kyoto Prefectural University of Medicine, Kawaramachi-Hirokoji, Kamigyo-ku, Kyoto 602-8566, Japan; katsura@koto.kpu-m.ac.jp (S.I.); shushi@koto.kpu-m.ac.jp (S.N.); kuniakih@koto.kpu-m.ac.jp (K.H.); tuchi-kf@koto.kpu-m.ac.jp (S.T.); hinoue@koto.kpu-m.ac.jp (H.I.); fjwr@koto.kpu-m.ac.jp (H.F.); s-shimo@koto.kpu-m.ac.jp (S.S.); tkubo@koto.kpu-m.ac.jp (T.K.); 2 Department of Sports and Para-Sports Medicine, Graduate School of Medical Science, Kyoto Prefectural University of Medicine, Kawaramachi-Hirokoji, Kamigyo-ku, Kyoto 602-8566, Japan; 3 Department of Immunology, Graduate School of Medical Science, Kyoto Prefectural University of Medicine, Kawaramachi-Hirokoji, Kamigyo-ku, Kyoto 602-8566, Japan; tsunao@koto.kpu-m.ac.jp (T.K.); masaharu@koto.kpu-m.ac.jp (M.S.-Y.); mazda@koto.kpu-m.ac.jp (O.M.)

**Keywords:** hypoxia-inducible factor 1 α, hyaluronic acid, CD44, articular cartilage, extracellular matrix

## Abstract

Hyaluronic acid (HA) is used clinically to treat osteoarthritis (OA), but its pharmacological effects under hypoxic conditions remain unclear. Articular chondrocytes in patients with OA are exposed to a hypoxic environment. This study investigated whether hypoxia could potentiate the anabolic effects of exogenous HA in rat articular cartilage and whether these mechanisms involved HA receptors. HA under hypoxic conditions significantly enhanced the expression of extracellular matrix genes and proteins in explant culture, as shown by real-time reverse transcription-polymerase chain reaction (RT-PCR), Western blotting, and dimethylmethylene blue (DMMB) assays. Staining with Safranin-O and immunohistochemical staining with antibody to type II collagen were also enhanced in pellet culture. The expression of CD44 was increased by hypoxia and significantly suppressed by transfection with siRNAs targeting hypoxia-inducible factor 1 alpha (siHIF-1α). These findings indicate that hypoxia potentiates the anabolic effects of exogenous HA by a mechanism in which HIF-1α positively regulates the expression of CD44, enhancing the binding affinity for exogenous HA. The anabolic effects of exogenous HA may increase as OA progresses.

## 1. Introduction

Osteoarthritis (OA) is a disease that negatively affects patients’ quality of life due to the irreversible progression of chronic pain and restriction of joint mobility [1]. Progressive degeneration of the articular cartilage is associated with age, injury, abnormal alignment, and metabolism disorders. Although the molecular mechanisms underlying cartilage degeneration caused by aging, mechanical stress, and inflammation have become clear, and risk factors for OA have been identified in a large-scale, population-based cohort study [2,3], curative drug therapy for OA has not yet been developed. Intra-articular injection has been considered to be important as a route of drug administration for OA so that the drug can reach the OA lesion directly. Several intra-articularly injected drugs that modify the pathogenesis of animal OA models have been reported recently. Interleukin-4 decreases nitric oxide production by chondrocytes and ameliorates subsequent destruction of cartilage in a rat OA model [4]. A hyaluronic acid-salmon calcitonin conjugate showed a chondro-protective effect in the local treatment of OA in a rabbit model of early OA [5]. Intra-articular injection of *N*-[*N*-(3,5-diflurophenylacetate)-l-alanyl]-(*S*)-phenylglycine t-butyl ester (DAPT), a small compound Notch inhibitor, in a surgical mouse OA model caused suppression of cartilage degradation [6]. These drugs have the potential as a curative agents against OA, but are not yet in clinical use.

High molecular weight hyaluronic acid (HA) has been widely used clinically as an intra-articular injection agent in the treatment of OA. As OA progresses, the molecular weight and concentration of HA in synovial fluid decrease, reducing the viscoelasticity of synovial fluid [7]. Viscoelasticity may be increased, however, following the intra-articular injection of high-molecular weight HA [8]. In addition, high-molecular weight HA has pharmacological effects. Exogenously administered high molecular weight HA has been reported to penetrate degenerated cartilage in patients with OA. This HA binds to HA receptors on the chondrocyte surface, such as CD44 and intercellular adhesion molecule-1 (ICAM-1), and inhibits matrix-degrading enzymes, such as matrix metalloproteinases (MMPs) [9].

Articular cartilage is an avascular tissue, with chondrocytes surviving in a hypoxic environment containing about 1%–5% oxygen [10]. The hypoxic environment surrounding articular chondrocytes in patients with OA has been reported to be due to increased oxygen usage by the synovial membrane, alterations in blood flow and gas exchange caused by fibrosis in the joint capsule, and subchondral bone sclerosis [11]. Although many studies assessing the in vitro effects of exogenous high molecular weight HA have been performed under standard normoxic conditions, the pharmacological effects and molecular mechanisms of exogenous HA under hypoxic conditions close to the actual physiological intra-articular environment have not been clarified.

Hypoxia-inducible factors (HIFs), which sense extracellular oxygen tension and regulate gene expression, play important roles as transcription factors in hypoxic environments [12]. HIF-1α is a transcription factor essential for chondrocyte differentiation and maturation, and for maintenance of chondrocyte phenotypes [13]. The expression of HIF-1α in OA cartilage also increases as articular cartilage degeneration progresses. Stress-induced HIF-1α may play an important role as a survival factor protecting tissue against catabolic changes during the progression of OA [14]. In contrast, HIF-1α has been found to affect sensitivity to anticancer drugs. In a hypoxic environment in tumor tissue, HIF-1α expression increases the potentiation of cell viability while inhibiting apoptosis [15]. Thus, the hypoxic environment of articular cartilage may also alter the pharmacological effects of intra-articularly injected drugs through the expression of HIF-1α.

Taking the above findings together, we hypothesize that hypoxic conditions may alter the pharmacological effects of exogenous high molecular weight HA on articular cartilage and chondrocytes. This study analyzed the ability of articular cartilage and chondrocytes cultured under hypoxic conditions to synthesize extracellular matrix (ECM) in the presence of exogenous HA and to determine the mechanisms of action of exogenous HA on primary articular chondrocytes under hypoxic conditions.

## 2. Results

### 2.1. Hypoxia with Hyaluronic Acid (HA) Significantly Potentiates the Synthesis of Extracellular Matrix (ECM)

**Figure 1:**
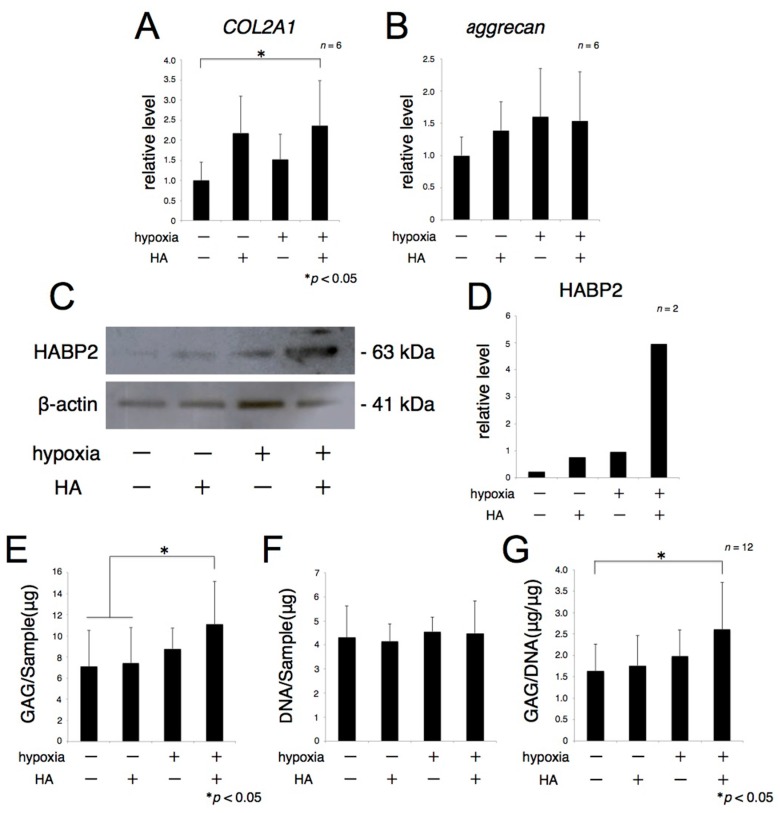
Exogenous hyaluronic acid (HA) promotes extracellular matrix (ECM) metabolism under hypoxic conditions. Rat articular cartilage was cultured in the presence of 4000 µg/mL HA for 72 h under hypoxia (1% oxygen). The expression of type II collagen and *aggrecan* mRNA was analyzed using real-time reverse transcription-polymerase chain reaction (RT-PCR) (**A**,**B**); and HA-binding protein 2 (HABP2) protein expression was analyzed by Western blotting (**C**,**D**); β-actin protein were used as loading controls. The total amount of glycosaminoglycans (GAG) in each sample was quantified using the dimethylmethylene blue (DMMB) assay (**E**); The DNA level was measured, and total GAG accumulation was normalized relative to DNA level (**F**,**G**). * *p* < 0.05.

To determine the metabolic effects of exogenous HA and hypoxia on the ECM, explants of rat articular cartilage were cultured for 72 h in the presence or absence of HA under normoxic or hypoxic conditions. Real-time RT-PCR showed that the level of *COL2A1* mRNA was significantly higher in explants treated with HA under hypoxic conditions than in the absence of HA under normoxic condition (Figure 1A). *Aggrecan* mRNA expression was also higher in explants treated with HA under hypoxic conditions than relative to its level in the absence of HA under normoxic conditions, but the difference was not statistically significant (Figure 1B). Western blotting showed that the level of HA-binding protein 2 (HABP2) was higher in explants treated with HA under hypoxic conditions (Figure 1C). Dimethylmethylene blue (DMMB) assays, measuring glycosaminoglycans (GAG) accumulation and DNA contents, were also performed to quantify cartilagenous ECM accumulation. The total GAG amount of each sample was slightly increased in the presence of HA under normoxic conditions and in the absence of HA under hypoxic conditions. In contrast, total GAG, and the ratio of GAG to DNA, were significantly increased in the presence of exogenous HA under hypoxic conditions (Figure 1D), although there were no changes in DNA contents among these four groups. These findings suggested that hypoxia plus exogenous HA promoted the accumulation of hyaline cartilagenous ECM, but not the proliferation of chondrocytes, present in explant cartilage samples (Figure 1E,F).

### 2.2. The Expression of Sex-Determining Region Y Box 9 (SOX9) Is Enhanced by Hypoxia with HA

**Figure 2:**
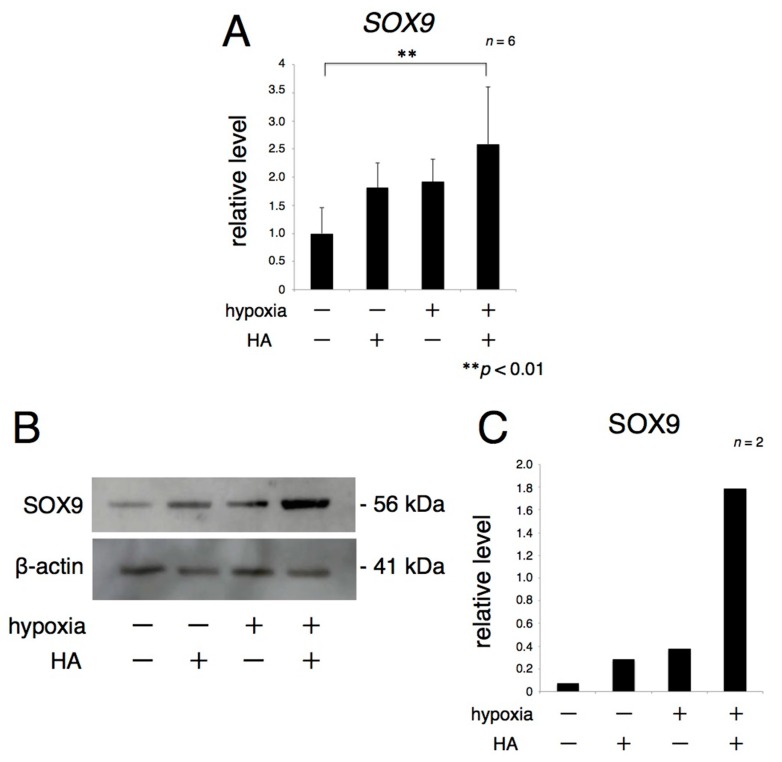
Exogenous HA promotes the expression of transcription factor upstream of ECM protein under hypoxic conditions. Rat articular cartilage was cultured in the presence of 4000 µg/mL HA for 72 h under hypoxia (1% oxygen). *SOX9* mRNA expression was analyzed by real-time RT-PCR (**A**); and SOX9 protein expression was analyzed by Western blotting (**B**,**C**). ** *p* < 0.01.

To assess the influence of exogenous HA on the expression of sex-determining region Y box 9 (SOX9), a transcription factor of ECM genes, explants of rat articular cartilage were cultured for 72 h in the presence or absence of HA under normoxic and hypoxic conditions. Real-time RT-PCR showed that the level of *SOX9* mRNA was increased in the presence of HA under both normoxic and hypoxic conditions and in the absence of HA under hypoxic relative to its level in the absence of HA under normoxic conditions (Figure 2A). Western blotting showed similar trends in protein expression (Figure 2B).

### 2.3. Hypoxia with HA Promotes the Redifferentiation of Rat Articular Chondrocytes (rACs) and the Synthesis of Hyaline Cartilagenous ECM

**Figure 3:**
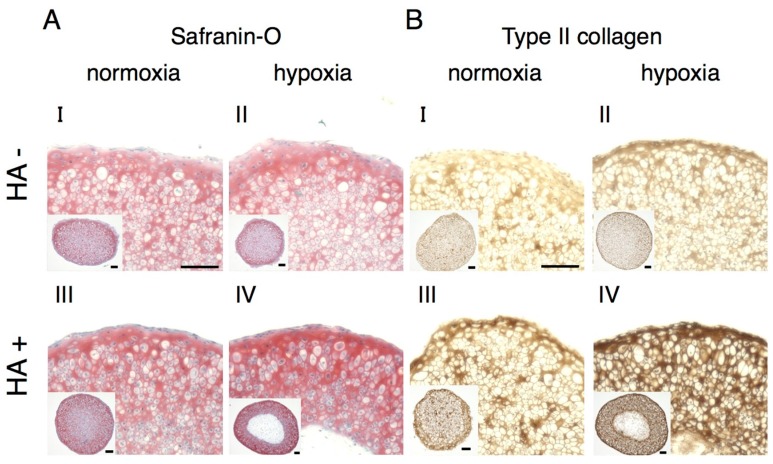
Exogenous HA promotes the redifferentiation of rat articular chondrocytes (rACs) and the synthesis of hyaline cartilage ECM under hypoxic conditions. Rat primary articular chondrocytes were subjected to 14 days pellet culture in the presence of 100 µg/mL HA under hypoxia (1% oxygen), followed by Safranin-O staining and type II collagen immunostaining (**A**,**B**). Scale bar = 100 µm.

To evaluate the effects of hypoxia plus exogenous HA on the redifferentiation of rat articular chondrocytes (rACs) and the synthesis of hyaline cartilagenous ECM, rACs isolated by treatment with collagenase were cultured as 3D pellet cultures for 14 days. The intensities of both of Safranin-O and type II collagen staining in cells cultured in the presence of HA under hypoxic conditions showed apparently stronger among these four groups (Figure 3AIV,BIV).

### 2.4. The Expression of CD44 Significantly Increases under Hypoxia

**Figure 4:**
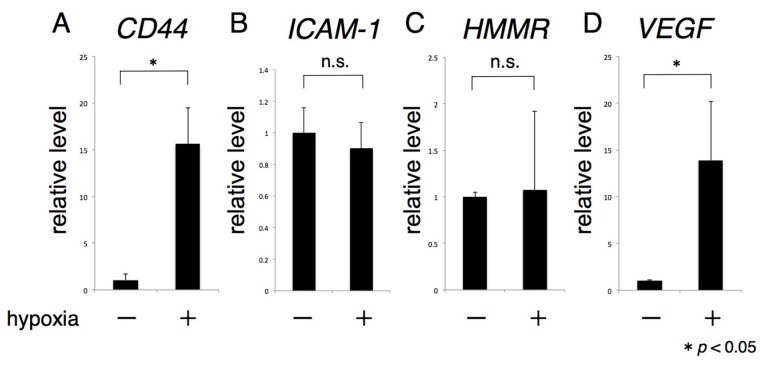
Hypoxia enhances the expression of *CD44*. Primary rACs were subjected to 72 h monolayer culture under hypoxia (1% oxygen), and the expression of various HA receptors was analyzed by real-time RT-PCR (**A**–**C**); as a positive control for the transcriptive activity of HIF-1α, *VEGF* mRNA expression was analyzed (**D**). * *p* < 0.05, n.s.: not significant.

*CD44* mRNA expression was significantly increased in rACs cultured under hypoxic, conditions, whereas the levels of other HA receptors, ICAM-1, and hyaluronan-mediated motility receptor (HMMR), were unchanged (Figure 4A–C). Expression of lymphatic vessel endothelial hyaluronan receptor-1 (LYVE-1) mRNA was not detected in articular chondrocytes (data not shown). The level of expression of *VEGF* mRNA, which is targeted by HIF-1α, was significantly increased by hypoxia, similarly to that of *CD44* mRNA (Figure 4D).

### 2.5. HIF-1α Controls CD44 Expression

**Figure 5:**
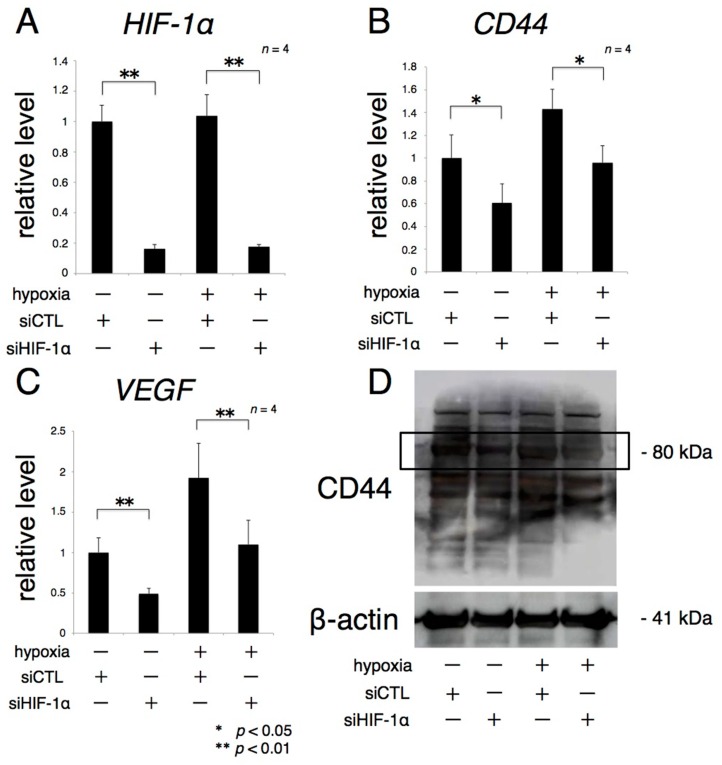
*CD44* expression is positively controlled by HIF-1α. Primary rACs transfected with siHIF-1α were grown for 72 h in monolayer cultures under normoxic and hypoxic (1% oxygen) conditions. Levels of *HIF-1α*, *CD44*, and *VEGF* mRNA were analyzed by real-time RT-PCR (**A**–**C**); CD44 protein expression was analyzed by Western blotting (**D**); β-actin was used as the loading control. * *p* < 0.05, ** *p* < 0.01.

More than 80% of the knockdown effect due to siHIF-1α on rACs persisted for 84 h after transfection under both normoxic and hypoxic conditions (Figure 5A). *CD44* and *VEGF* mRNA expression, which were increased by hypoxia, were significantly reduced by transfection of siHIF-1α (Figure 5B,C). Similarly, CD44 protein expression was inhibited by transfection of siHIF-1α focusing on the bands around 80 kDa: predicted molecular weight of CD44 (Figure 5D, black lined frame). The intensity of the other bands considered to be possible isoforms was also decreased by transfection of siHIF-1α [16].

## 3. Discussion

This report showed that hypoxic conditions positively potentiate the anabolic effects of exogenous high molecular weight HA, as shown by ECM synthesis and the redifferentiation of articular cartilage and chondrocytes. These findings supported our hypothesis stated in the introduction. The mechanism by which hypoxia potentiates these effects may include increased CD44 expression and increased binding affinity to exogenous high molecular weight HA.

Exogenous high molecular weight HA has been reported to promote GAG synthesis in chondrocytes under normoxic conditions [17,18]. Exogenous high molecular weight HA can infiltrate articular cartilage in normal individuals and patients with OA, binding to HA receptors expressed on the chondrocyte surface [9]. Compared with normoxia, hypoxia has been reported to increase the synthesis of GAG and type II collagen synthesis in articular chondrocytes [19] and to simultaneously inhibit matrix-degrading enzymes, such as MMP-13 [20]. We also previously reported that hypoxia enhanced the expression of proteoglycan and type II collagen genes in chondrocytes [21]. In addition, culture of human normal and OA chondrocytes cultured under hypoxic conditions was found to increase the expression of HAS2 [22]. Although exogenous high molecular weight HA and hypoxia have been individually reported to enhance cartilage matrix metabolism, their combined effects had not been determined. In this study, articular cartilage explants were cultured under hypoxic conditions in the presence of exogenous HA to examine their effects on the synthetic ability of hyaline cartilagenous ECM. The amount of GAG and the expression of *COL2A1* mRNA was significantly higher in explants cultured under hypoxic conditions in the presence of HA than under normoxic conditions in the absence of HA. The expression of *aggrecan* mRNA and the protein production of HABP2 which is an ECM protein was also increased. These findings suggested that ECM production was greater in the presence of both exogenous high molecular weight HA and hypoxia than either alone, and support our original hypothesis stated in the introduction. Normal articular chondrocytes survive physiologically under hypoxic conditions and OA chondrocytes are also exposed to a greater degree of hypoxia. These findings also indicated that hypoxic environments may play an important role in potentiation of ECM synthesis stimulated by exogenous high-molecular weight HA in vivo.

It is well known that primary articular chondrocytes easily lose their specific phenotypes after long expansion time and multiple passaging in monolayer culture, which is referred to as “dedifferentiation”. Dedifferentiation of chondrocytes is defined as the gradual loss of molecular markers such as type II collagen and aggrecan and the acquisition of a fibroblastic phenotype such as type I collagen at the same time [23]. Dedifferentiated articular chondrocytes result in regain of a differentiated articular chondrocyte phenotype and re-expression of hyline cartilage marker molecules by high cell density three-dimensional (3D) cultures, which is called “redifferentiation” [24,25]. Although it was reported that hypoxic conditions promote redifferentiation of dedifferentiated articular chondrocytes cultured in monolayer expansion and ECM synthesis of redifferentiated articular chondrocytes cultured in 3D pellet [20], the effects of exogenous high-molecular weight HA on redifferentiation and ECM synthesis of articular chondrocytes has remained unclear. In the present study, pellet cultures grown under hypoxic conditions in the presence of HA showed strong intensities of Safranin-O and type II collagen staining demonstrating, histologically, that exogenous high molecular weight HA promotes redifferentiation and ECM synthesis of articular chondrocytes under hypoxic conditions.

SOX9 is a transcription factor directly controlling type II collagen expression [26], as well as being important for differentiation into mature chondrocytes and their maintenance [27]. SOX9 is partly controlled by HIF-1α during early skeletal formation [28]. However, the effects of exogenous high-molecular weight HA on SOX9 expression in mature articular cartilage under hypoxic conditions had not been previously determined. We found that SOX9 expression was higher in explants cultured in the presence of HA and hypoxia than in the presence of HA and normoxia or hypoxia alone, suggesting that the combination of exogenous high-molecular weight HA and hypoxia may maintain hyaline cartilage and promote production of ECM of chondrocytes by activating the transcription of SOX9.

In assessing the mechanisms of action of exogenous HA and hypoxia, we focused on the expression of HA receptors. Several types of HA receptor have been identified, including CD44, ICAM-1, HMMR, and LYVE-1 [29,30,31,32], but the effects of hypoxia on their levels of expression in cultured chondrocytes had not been determined. Although hypoxia had little effect on the expression of *ICAM-1*, *HMMR*, and *LYVE-1* mRNAs, it markedly enhanced the expression of *CD44*, as well as of *VEGF*, mRNAs. Knockdown of HIF-1α in hypoxic culture significantly suppressed the expression of CD44 and *VEGF* mRNA and protein. CD44 expression has been reported higher in breast cancer stem cells under conditions of hypoxia than normoxia, and HIF-1α was found to control CD44 expression in these cells [33]. Our findings suggest that HIF-1α also controls CD44 expression in articular chondrocytes, altering the binding affinity of CD44 and exogenous HA and the pharmacological effects of exogenous HA.

This study had several limitations, including the culture methods. Explant, 3D pellet, and monolayer cultures were tested, depending on the factors of interest and the analytic methods. However, the findings in these cultures were consistent with each other. Another limitation was our inability to distinguish between chondrocytes in the superficial and deep layers because we used rats and harvested full-thickness articular cartilage.

## 4. Materials and Methods

### 4.1. Ethics Statement

This study was conducted according to the regulations regarding animal research of Kyoto Prefectural University of Medicine (Code no. M26-29).

### 4.2. Cartilage Samples

Male Wistar rats, aged six weeks (Shimizu Laboratory Supplies, Kyoto, Japan), were sacrificed by cervical dislocation, and articular cartilage was aseptically collected from their knee, hip, and shoulder joints. The specimens from each rat were pooled and cut into small cubes, about 1 mm per side, for explant culture. Samples were cultured in 12-well culture plates (Greiner Bio-One, Frickenhausen, Germany), with each well containing 2 mL of Dulbecco’s modified Eagle’s medium (DMEM; Nacalai Tesque, Kyoto, Japan) supplemented with 10% fetal bovine serum (FBS; Equitech-Bio, Kerrville, TX, USA), 100 units/mL penicillin, and 100 mg/mL streptomycin (complete DMEM; Nacalai Tesque, Kyoto, Japan) overnight at 37 °C in 5% CO_2_/95% humidified air.

### 4.3. Isolation and Culture of Rat Articular Chondrocytes (rACs)

Articular cartilage specimens, collected and minced into small pieces as above, were incubated with 0.015% trypsin (Sigma-Aldrich, St. Louis, MO, USA) for 15 min, and subsequently digested with 0.25% collagenase (Collagenase L; Nitta Gelatin, Osaka, Japan) for 4 h at 37 °C [34]. The isolated chondrocytes were cultured as monolayers in 75 cm^2^ flasks (Falcon, Lincoln, NY, USA) in complete DMEM for one week at 37 °C in 5% CO_2_/95% humidified air.

### 4.4. 3D Pellet Culture of rACs

Primary rACs (10^5^ cells/well) were inoculated into V-bottom 96-well plates (Sumitomo Bakelite, Tokyo, Japan), centrifuged at 1000 rpm for 5 min and incubated for 14 days with 200 µL of complete DMEM [35]. Medium was changed before subsequent experiments.

### 4.5. Hyaluronic Acid Treatment and Hypoxia Experiments

Articular cartilage explant cultures were washed twice with PBS, followed by incubation for 72 h at 37 °C with 2 mL of complete DMEM containing 0 or 4000 mg/mL hyaluronic acid (*M*_W_ = 800 kDa, Seikagaku Corp., Tokyo, Japan) under normoxic or hypoxic (1% O_2_ or 5% CO_2_, and 94% N_2_) conditions in a multigas incubator (MODEL 9200, Wakenyaku Co., Ltd., Kyoto, Japan). 3D pellet cultures of rACs were incubated for 14 days at 37 °C with 200 µL of complete DMEM containing 0 or 100 mg/mL hyaluronic acid under normoxic or hypoxic conditions, as above. Media were changed every three days.

### 4.6. Transfection with Small Interfering RNA (siRNA)

Primary rACs were seeded into 24-well plates at a density of 3 × 10^4^ cells per well at 37 °C in 5% CO_2_/95% humidified air (normoxic conditions). The next day, the medium was changed and rACs were transfected with validated MISSION HIF-1α siRNA (SASI_Rn01_00053994; Sigma-Aldrich) or a negative control (MISSION siRNA Universal Negative Control; SIC-001; Sigma-Aldrich) at final concentrations of 50 nM in cationic liposome (Lipofectamine RNAiMAX Reagent; Invitrogen, Carlsbad, CA, USA) according to the manufacturer’s instructions. The plates were cultured under standard normoxic conditions for 12 h. The medium was changed, and the cells incubated for 72 h under normoxic or hypoxic conditions.

### 4.7. Total RNA Extraction and Real-Time Reverse Transcription-Polymerase Chain Reaction (RT-PCR) Analysis

Explants frozen in liquid nitrogen were washed twice with PBS and physically crushed using a Cryo-Press CP-100WP (Microtech Nichion, Chiba, Japan). Crushed explants were harvested and total RNA was extracted from these cells using ISOGEN (Nippon Gene, Osaka, Japan). Extracted RNAs were reverse transcribed using a PrimeScript^TM^ RT Master Mix (Takara Bio, Shiga, Japan) according to the manufacturer’s instructions. Quantitative real-time RT-PCR was performed using a Biosystem 7300 (Applied Biosystems, Carlsbad, CA, USA) with TaqMan Assay-on Demand gene expression primer/probe sets (Applied Biosystems) for *COL2A1* (Rn01637087_m1), *ACAN* (Rn00573424m1), *SOX9* (Rn01751069_mH), *CD44* (Rn00563924_m1), *ICAM1* (Rn00564227_m1), *HMMR* (Rn00564204_m1), *VEGF* (Rn01511602_m1), and *HIF1A* (Rn01472831_m1). Each 25 µL reaction mixture contained 2 µL of cDNA (100 ng), and 12.5 µL TaqMan Gene Expression PCR Master Mix (TOYOBO, Osaka, Japan) for the target gene. The amplification protocol consisted of 40 cycles of denaturation at 95 °C for 15 s and annealing and extension at 60 °C for 1 min. Relative changes in gene expression were calculated according to the comparative CT method and normalized against the internal control gene, 18S ribosomal RNA (forward primer, 5′-ATGAGTCCACTTTAAATCCTTTAACGA-3′; reverse primer, 5′-CTTTAATATACGCTATTGGAGCTGGAA-3′; probes, 5′-(FAM)ATCCATTGGAGGGCAAGTCTGGTGC(BHQ)-3′). Results were the means of three or four samples, with each sample assayed in duplicate.

### 4.8. Protein Extraction

Explants frozen in liquid nitrogen, washed twice with PBS and physically crushed using a Cryo-Press CP-100WP (Microtech Nichion), were harvested and lysed in 300 µL per well of RIPA buffer diluted 10-fold (Nacalai Tesque, Kyoto, Japan). For chondrocyte monolayer cultures, the cells were washed twice with PBS and lysed in RIPA buffer, as above. Samples were frozen at −80 °C for 10 min, thawed at 37 °C, and incubated on ice for 30 min. The samples were centrifuged at 15,000 rpm for 5 min and the supernatant was aspirated.

### 4.9. Western Blot Analyses

Protein concentrations of samples were measured using bicinchoninic acid (BCA) assays, and samples containing equal volumes of lysis buffer from an equal number of similarly-sized explants or chondrocytes were loaded onto gels for sodium dodecyl sulfate polyacrylamide gel electrophoresis (SDS-PAGE). Cartilage and cell extract samples containing 20 µg of protein were separated by 10% Bis-Tris Gel NuPAGE^®^ electrophoresis using 5% MOPS SDS Running Buffer (Novex, CA, USA). Separated proteins were dry blotted onto nitrocellulose iBlot^®^gel transfer stacks in iBlot Gel transfer devices for 7–10 min. The nitrocellulose membrane was blocked by shaking in Blocking One (Nacalai Tesque, Kyoto, Japan) for 60 min at room temperature. The blots were subsequently shaken overnight at 4 °C in solution containing the following specific primary antibodies: mouse anti-HIF-1α (1:500 dilution; Novusbio catalog no. NB100-105), rabbit anti-SOX9 (1:1000 dilution; Abcam catalog no. ab185230), rabbit anti-HABP2 (1:10,000 dilution; Abcam catalog no. ab181837), and rabbit anti-CD44 (1:200 dilution; Abcam catalog no. ab24504), and with mouse anti-β-actin (1:4000 dilution; Sigma-Aldrich catalog no. A2228) as loading controls. Blots were washed three times with Tris Buffered Saline with Tween 20 (TBST) and shaken for 60 min at room temperature with peroxidase-conjugated anti-mouse IgG (1:4000 dilution; Sigma-Aldrich catalog no. A4416) or peroxidase-conjugated anti-rabbit IgG (1:4000 dilution; Sigma-Aldrich catalog no. A0545) secondary antibodies, as appropriate. The blots were again washed three times with TBST, and chemiluminescence emission was visualized using Chemi-Lumi One L (Nacalai Tesque). Protein band intensities were assessed using an ECL Select LAS500 (GE Healthcare). Band densities were quantified using Image J software (National Institute of Health, Bethesda, MD, USA), and presented as relative level to β-actin which is the internal control. The same assay as above was conducted in two independent experiments.

### 4.10. Biochemical Analyses

Dimethylmethylene blue (DMMB) assays quantifying the amount of glycosaminoglycans (GAG) in cartilage explants were performed using Proteoglycan Detection Kits (Rheumera^TM^; Cat# 8000, Astarte Biologics, Bothell, WA, USA) according to the manufacturer’s instructions. Briefly, explants frozen in liquid nitrogen were washed twice with PBS and physically crushed using a Cryo-Press CP-100WP (Microtech Nichion, Chiba, Japan). The crushed cartilage explants were digested in 20 mM sodium phosphate buffer (pH 6.8) containing 1 mM EDTA, 2 mM dithiothreitol, and 600 µg/mL Papain (Nacalai Tesque), at 60 °C for 2 h, followed by the addition of 50 mM Tris/HCl (pH 8.0) containing 10 mM iodoacetic acid (Nacalai Tesque). The metachromatic reaction of GAG with DMMB was monitored spectrophotometrically at 525 nm [36]. The total amount of GAG was normalized to the total amount of DNA in the same sample.

### 4.11. Histological and Immunohistochemical Analyses

Pellets were fixed in 4% paraformaldehyde (Wako, Osaka, Japan), embedded in paraffin and cross-sectioned (5 µm thick sections). The sections were stained with Safranin-O to detect sulfated GAG and processed for immunohistochemistry to visualize type II collagen (F-57, Daiichi Fine Chemical, Toyama, Japan).

### 4.12. Statistical Analysis

All duplicate and triplicated experiments yielded almost identical results. All experimental data are expressed as mean ± standard deviation (SD). Parametric one-way analysis of variance (ANOVA) was used to test for any differences among the groups. If the result was significant, the Tukey-Kramer test was used to determine the specific differences between groups. In all analyses, *p* < 0.05 was defined as statistically significant.

## 5. Conclusions

To our knowledge, this study is the first to show that hypoxia promotes the anabolic pharmacological effects of exogenous high molecular weight HA on articular cartilage. The mechanism may involve HIF-1α, which may enhance CD44 expression and the binding affinity of CD44 and exogenous high-molecular weight HA. Hypoxic conditions around articular chondrocytes play an important role in the anabolic pharmacological effects of exogenous HA. HIF-1α expression in OA cartilage has been reported to correlate with the progression of articular cartilage degeneration [14]. The pharmacological effects of exogenous high molecular weight HA may also correspond to the progression of OA.
